# Hypofractionated whole breast irradiation with simultaneous integrated boost in breast cancer using helical tomotherapy with or without regional nodal irradiation: A report of acute toxicities

**DOI:** 10.3389/fonc.2023.1122093

**Published:** 2023-03-17

**Authors:** Imjai Chitapanarux, Wannapha Nobnop, Wimrak Onchan, Pitchayaponne Klunklin, Thongtra Nanna, Chomporn Sitathanee, Sutthisak Kulpisitthicharoen, Patumrat Sripan

**Affiliations:** ^1^ Division of Radiation Oncology, Faculty of Medicine, Chiang Mai University, Chiang Mai, Thailand; ^2^ Northern Thai Research Group of Radiation Oncology (NTRG-RO), Faculty of Medicine, Chiang Mai University, Chiang Mai, Thailand; ^3^ Division of Radiation Oncology, Faculty of Medicine, Ramathibodi Hospital, Bangkok, Thailand; ^4^ Division of Radiation Oncology, Lopburi Cancer Hospital, Lopburi, Thailand; ^5^ Research Institute for Health Sciences, Chiang Mai University, Chiang Mai, Thailand

**Keywords:** regional nodal irradiation (RNI), simultaneous integrated boost (SIB), hypofractionation, breast cancer, helical tomotherapy (HT), acute toxicities

## Abstract

**Purpose:**

We prospectively investigated the acute toxicities focusing on skin and hematologic function in breast cancer patients who received hypofractionated whole breast irradiation with simultaneous integrated boost (HF-WBI-SIB) with helical tomotherapy (HT), with or without regional nodal irradiation (RNI).

**Methods:**

The dose of WBI and RNI was 42.4 Gy in 16 fractions. Tumor bed was prescribed to 49.6 Gy in 16 fractions simultaneously. The association between the worst grade of acute toxicities during treatment and receiving RNI was analyzed. The integral dose to the whole body between the two groups was also compared.

**Results:**

Between May 2021 and May 2022, 85 patients were enrolled; 61 patients received HF-WBI-SIB only (71.8%) and 24 patients (28.2%) received HF-WBI-SIB with RNI. Grade 2 acute skin toxicity was found in 1.2%. The most frequent grade 2 or more hematologic toxicity was leukopenia, which occurred in 4.8% and 11% in the 2nd and 3rd week, respectively. Mean whole body integral dose was significantly higher in patients treated with RNI compared to patients treated without RNI: 162.8 ± 32.8 *vs*. 120.3 ± 34.7 Gy-L (p-value < 0.001). There was no statistically significant difference in acute grade 2 or more skin and hematologic toxicities between the two groups.

**Conclusions:**

HF-WBI-SIB with or without RNI is feasible with acceptable acute skin and hematologic toxicities. RNI and whole body integral dose were not associated with these acute toxicities.

## Introduction

Radiotherapy (RT) plays an important role in the treatment of breast cancer patients undergoing breast-conserving surgery (BCS). RT improves both local control and breast cancer specific survival as shown by a meta-analysis of 17 trials, most of them using conventional fractionation (1). Phase III randomized trials investigating hypofractionated (HF) dose delivery for whole breast irradiation (WBI) demonstrated equivalence with conventional fractionation (CF) both in clinical outcomes and toxicity profiles (2, 3).

Our previous study of HF radiotherapy using Helical TomoTherapy (HT) to the chest wall/breast with/without regional nodal irradiation (RNI) demonstrated excellent 3-year locoregional failure-free survival (LRFFS) and minimal acute and late toxicities (4). HF with simultaneous integrated boost (SIB) has been studied and seems to be practical and safe (5). A phase II study using Volumetric Modulated Radiation Therapy (VMAT) for hypofractionated whole breast irradiation with simultaneous integrated boost (HF-WBI-SIB) confirmed the safety and reported good cosmetic results, even in patients who received adjuvant systemic therapy (5, 6). The latest comparative dosimetric study (7), demonstrated that HF-WBI-SIB using HT with TomoEdge offered a significantly lower mean equivalent dose in 2-Gy fractions (EQD2) to OARs and showed no significant difference between HF-SIB and sequential boost. Both HF-SIB and normally fractionated SIB (N-SIB) conformed significantly better to the breast and boost planning target volumes (PTV) than both sequential boost techniques.

HT, a fan beam intensity modulated radiotherapy (IMRT) technique, characterized by a helical movement of the beam delivery, provides satisfactory target coverage and doses to the surrounding organs at risk (OARs). However, it can cause a larger volume of normal tissue in the treated area and whole body to receive low radiation doses as a result of the longer beam on times (8). The increase in normal tissue integral dose caused by IMRT has given concern for radiation-induced secondary malignancies (9). Studies on this issue in patients treated with HT show mixed results: some have found an increase of the integral dose with HT (10, 11) while others found contradicting results with no increase of the integral dose with HT as compared to conventional IMRT techniques, and in some cases even a slight decrease (12, 13).

To our knowledge, all previous HF-WBI-SIB trials excluded patients who needed RNI. We conducted a prospective multicenter study on HF-WBI-SIB in breast cancer patients after breast conserving surgery treated by HT, to which we also added RNI when indicated. In the present report, we assess the acute toxicities and calculate the integral dose to the whole body in breast cancer patients who received HF-WBI-SIB with or without RNI using HT. The association between integral dose and acute toxicities is also explored. Treatment outcome, which includes field boost recurrence (IFBR) rate (tumor recurrence in boost area), locoregional recurrence (LRR) rate (tumor recurrent in ipsilateral breast and/or regional lymph node area), cosmetic results, and late toxicities will be reported after a longer follow-up.

## Material and methods

### Patients

This phase II prospective study was registered with the Thai Clinical Trials Registry (TCTR20210623004) and was approved by the institutional review board at each of the three contributing centers: Chiang Mai University (Chiang Mai), Ramathibodi Hospital (Bangkok), and Lopburi Cancer Hospital (Lopburi). All patients provided written informed consent. Eligible patients were patients who received breast conserving surgery (BCS) for a pathologically confirmed invasive ductal carcinoma, had surgical margins free from both invasive carcinoma and ductal carcinoma *in situ* (DCIS), were age ≥ 18 years, with ECOG performance status 0 or 1. All patients had an indication for tumor bed boost according to institutional protocols (age < 50 years or age > 50 years with high-risk features). The tumor bed had to be clearly identified (preferred by radiopaque clips). Patients who needed regional nodal irradiation (RNI) were allowed in this study. We excluded patients who had bilateral breast cancer, had extensive postoperative seroma at the commencement of RT, and patients who met all the following criteria: age > 70, T1, N0, ER+, low-intermediate grade, margin ≥ 2mm.

### Radiotherapy

All patients underwent a three-dimensional simulation procedure in the supine position on a wing board (CIVCO, USA) with both arms up above the head. Computed tomography (CT) was performed with a slice thickness of 5 mm, using radiopaque wires to define the scars and field borders on the patients’ skin during CT-simulation. The entire mammary gland constituted the clinical target volume (CTV) of the whole breast (WB). The tumor bed plus 1 cm added to the surgical clips placed in the lumpectomy cavity constituted the CTV of the boost. A five-millimeter margin was added to form the planning target volumes (PTV) for each of these CTVs. The ribs and lung tissue were excluded from the PTV. To reduce the potential for skin reactions and dose inhomogeneity, breast PTV was restricted to a depth of 3 mm under the skin surface. We followed the Radiation Therapy Oncology Group (RTOG) atlas to contour CTV/PTV (14). We prescribed the radiation dose with the SIB technique for 16 fractions with 2.65 Gy/fraction to a total dose of 42.4 Gy for the PTV WB, and 3.1 Gy/fraction to a total dose of 49.6 Gy to the PTV boost. For the patients who received RNI, the PTV for the lymph nodes was separated and prescribed at 2.65 Gy/fraction to a total of 42.4 Gy. HT treatment plans were created using a jaw width of 5.0 cm, a pitch of 0.287, and a modulation factor of 3.0. We created a directional block to limit the entrance dose to the following OARs: the bilateral lungs, the contralateral breast, the heart, and the left anterior descending coronary artery (LAD). Plan objectives, concerning target coverage and homogeneity, were as follows: near-to-minimum dose D _98%_ >95%, near-to-maximum dose D _2%_ <107% for PTV WB and RNI (where D _x%_ was the dose delivered to at least or at most x %). The dose parameters for OARs are shown in **Table 1** where Vx_Gy_ was the volume receiving at least xGy. Integral dose, which is the volume integral of the dose deposited in each patient, was explored. The whole body integral dose is defined as the mean dose in Gray (Gy) of the entire volume of all slices where PTV existed plus 2 cm superior and inferior to the PTV, multiplied by the volume of the whole body in Liter (L) (13, 15).

**Table 1:** Dose constraint for organ at risk (OARs) in this study.

OAR	Acceptable	
	WBI	WBI + RN
Heart (Right Breast)	D_max_ < 20 Gy V_8Gy_ < 15%	D_15%_ < 10 Gy D_20%_ < 8 Gy D_mean_ < 9 Gy
Heart (Left Breast)	D_5%_ < 20 Gy V_8Gy_ < 35%	D_15%_ < 10 Gy D_20%_ < 8 Gy D_mean_ < 9 Gy
Left anterior descending coronary artery (LAD)	–	D _mean_ < 9.7 Gy D_1%:_ < 16.1 Gy
Ipsilateral lung	V_16Gy_ < 20% V_8Gy_ < 40%	D_15%_ < 31 Gy D_20%_ < 26.4 Gy D_35%_ < 17.6 Gy D_50%_ < 13 Gy
Contralateral lung	V_4Gy_ < 15%	D_20%_ < 13 Gy D_35%_ < 10.6 Gy D_50%_ < 9 Gy
Contralateral breast	D_max_ < 2.64 Gy	D_15%_ < 17.6 Gy D_20%_ < 9 Gy D_35%_ < 6 Gy D_50%_ < 4.4 Gy
Esophagus	–	D_max_ < 15 Gy

Radiotherapy started within 6 weeks after the last dose of chemotherapy. Clinical assessment for acute skin and hematologic toxicities were assessed once a week during RT using the RTOG/EORTC acute radiation morbidity score (16). Our endpoints of interest were the worst grade of acute skin toxicity and the nadirs of white blood cell (WBC), hemoglobin and platelets, defined as the least value occurring between the start of RT and the end of RT.

### Statistical analysis

A descriptive analysis was performed to calculate proportions and frequencies of patient and treatment characteristics, while medians with interquartile ranges (IQR) were calculated for the age of patient. A mean with standard deviation (SD) was calculated for the integral dose and dosimetric characteristics. Acute skin and hematologic toxicities were assessed as frequency and percentages per grade. The association between the worst grade of acute toxicities during treatment (week 1, week 2 and week 3) and receiving RNI, considered as a binary variable was analyzed using the Fisher exact test. The Wilcoxon-Mann-Whitney test was used to compare the integral dose and dosimetric characteristics between groups of patients who received HF-WBI-SIB using HT, with or without RNI respectively. Analyses were performed using STATA software version 16 (Stata Corp, College Station, TX).

## Results

Between May 2021 and May 2022, 85 patients were enrolled from 3 radiotherapy centers: 61 patients received HF-WBI-SIB only (71.8%) and 24 patients (28.2%) received HF-WBI-SIB with RNI. The median age was 53 years (IQR: 45-59, Range: 32-73). Over 90% of patients had stage I and II disease and only 9% had stage III disease. HER2-/HR+ was the most common subtype (64.7%) followed by HER2+/HR+, HER2 enriched, and triple negative. Most of the patients in this study had received previous adjuvant chemotherapy (85.9%) with an anthracycline-based regimen. There was no statistically significant difference in the mean PTV volume between patients who received HF-WBI-SIB without RNI (824.4 ± 339.8 cm^3^) and those with RNI (991.5 ± 494.1 cm^3^), p= 0.12. The whole body integral dose in the group of patients receiving HF-WBI-SIB with RNI was significantly higher than the group without RNI (p- value <0.001). The patients and treatment characteristics are summarized in **Table 2**.

**Table 2:** Patient and treatment characteristics.

Variables	N (%)
Age (years) Median = 53 (IQR: 45-59, Range: 32-73)	
<40	11 (12.9)
41-50	25 (29.4)
51-60	31 (36.5)
>60	18 (21.2)
Smoking	
YES	0 (0.0)
NO	85 (100.0)
BMI (kg/m2)	
<18.5	6 (7.0)
18.5-24.9	48 (56.5)
25-29.9	18 (21.2)
>30	13 (15.3)
Underlying cardiac disease	
YES	6 (7.0)
NO	79 (93.0)
AJCC stage	
I	37 (43.5)
II	40 (47.1)
III	8 (9.4)
Subtype	
HER2-/HR+	55 (64.7)
HER2+/HR+	14 (16.5)
HER2+/HR−	5 (5.9)
HER2−/HR−	11 (12.9)
Chemotherapy	
YES	73 (85.9)
AC4	41 (56.1)
FAC6	8 (11.0)
AC4T4	24 (32.9)
NO	12 (14.1)
Hormonal therapy	
YES	67 (78.8)
Tamoxifen	42 (62.7)
Aromatase inhibitor	25 (37.3)
NO	18 (21.2)
Regional nodal irradiation (RNI)	
YES	24 (28.2%)
NO	61 (71.8%)
PTV Volume (Mean ± SD) (cm3)	
Breast only	824.4 ± 339.8
Breast + RNI	991.5 ± 494.1
Whole body Integral dose (Mean ± SD) (Gy-L)	
Breast only	120.3 ± 34.7
Breast + RNI	162.8 ± 32.8

Regarding the target coverage, following the International Commission on Radiological Units and Measurements (ICRU) no. 83, all plans were approved when the near-to-maximum dose D _2%_ was less than 53.1 Gy (107% of the prescription for PTV boost) and the near-to-minimum dose D _98%_ was more than 47.1 Gy (95% of the prescription for PTV boost) and 40.3 Gy (95% of the prescription for PTV WB), respectively.

Pre-radiotherapy hematological data of our patients was recorded. All patients had no thrombocytopenia. One patient (1.2%) had grade 1 anemia, while grade 1, 2, and 3 leukopenia were found in 9 patients (10.6%), 1 patient (1.2%), and 1 patient (1.2%), respectively.

**Figure 1:**
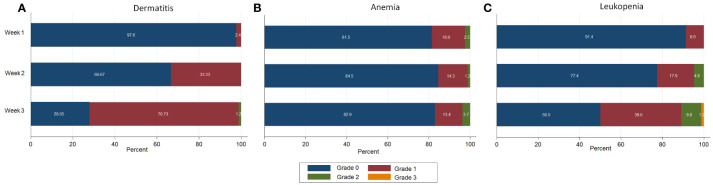
Acute toxicities during treatment in all patients. **(A)** Dermatitis **(B)** Anemia **(C)** Leukopenia.

**Figure 2:**
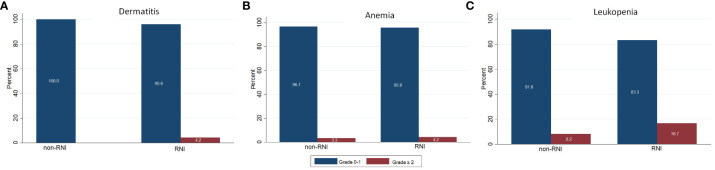
Comparing of acute toxicities between patients received and not received regional nodal irradiation. **(A)** Dermatitis **(B)** Anemia **(C)** Leukopenia.

We found that the compliance of this RT scheme was very good: all patients could complete their treatment. Grade 2 acute skin toxicity was found in 1 patient (1.2%) during the 3^rd^ week of treatment. The most frequent hematologic toxicity was leukopenia. We found grade 2 leukopenia in 4 patients (4.8%) during the 2^nd^ week, which increased to 9.8% in the 3^rd^ week of treatment. Grade 3 leukopenia was demonstrated in 1 patient (1.2%) at the 3^rd^ week, for this patient the treatment needed to be delayed. **Figure 1** shows the acute skin and hematologic toxicity during the 3 weeks of treatment in all patients. There was no statistically significant difference in both acute severe (grade ≥2) skin and hematologic toxicities between patients who received RNI and those who did not, as shown in **Figure 2**.

## Discussion

Our present multicenter prospective phase II study, using HF-WB-SIB for 16 fractions with 2.65 Gy/fraction to a total dose of 42.4 Gy for the PTV WB and RNI, and 3.1 Gy/fraction to a total dose of 49.6 Gy to the PTV boost, revealed acceptable rates of acute skin and hematologic toxicities. HF-WB-SIB (without RNI) was investigated in many prospective studies and reported satisfactory result on early acute toxicities. A prospective phase III randomized controlled trial by Paelinck et al. (17) compared the acute toxicities between HF-WBI with a sequential boost (40.05 Gy/15 fractions + 10 Gy/4 fractions in negative surgical margins or 14.88 Gy/6 fractions in positive margins) or SIB (42.4/46.8 and 49.95 Gy/15 fractions in negative and positive margin, respectively) planning by VMAT and irradiated in prone position. They also reported that HF-WBI-SIB had significantly lower grade 2/3 dermatitis and pruritus. Focusing only on their SIB arm, grade 2 or 3 dermatitis was found in 24/83 patients (28%) which was much higher than in our study which reported grade 2 in only 1.2% and no grade 3 toxicity. When considering the circumstances, the PTV volume of their study did not differ from our PTV volume (in the breast without RNI group). We hypothesize that the prone position in their study could be the cause of more severe skin toxicity, compared to the supine position. However, the authors also indicated a limitation in their scoring of toxicity, which relied on subjective grading.

A phase I/II study from India (18) performed HF-WBI-SIB with 40.5 and 48 Gy in 15 fractions with VMAT in 10 patients. They reported satisfactory PTV coverage and OAR sparing. The most common acute toxicities were grade 1 dermatitis. Grade 2 skin toxicities were found in 2 patients (20%). This study had also higher grade 2 acute skin toxicity than ours. This might due to the small number of patients and the fact that the mean volume of PTV whole breast and boost was higher than in our patients (1015.08 cm^3^ versus 824.4 cm^3^ in our breast without RNI group). Their margin of CTV boost was an additional 1.5 cm margin from the surgical bed, whereas 1 cm was used in our study. VMAT-SIB hypofractionation was investigated by De Rose et al. (5). They reported 8% of grade 2 RTOG acute toxicity which were found in the last week of treatment, which is comparable to our findings. However, no grade 2 patient in their study had moist desquamation while we found this in 1 patient (1.2%). The latest multicenter prospective phase II study from Germany (RO-2013-04, NCT01948726) (19) reported the outcome of HF-WBI-SIB using 40/48 Gy in 16 fractions. Grade 2 or more skin toxicity was found in 14.7% which was also higher than our study. More than half of the patients (58.7%) in this study received 3D-CRT, which could be an explanation for the increased occurrence of toxicities.

As a consequence of enrolling the patients who need RNI in our study (28.2%), the percentage of patients who had prior chemotherapy before RT in our study was the highest (85.9%) when compared to 34% in a German trial (19) and 32% in a Belgian study (17). Almost of our patients (85.9%) received anthracycline-based chemotherapy which has myelosuppression as a side effect. Eleven patients (13.0%) had leukopenia and 1 patient (1.2%) had anemia before starting radiotherapy. However, we found that the number of acute grade 2 or more hematologic toxicities was still increasing during the treatment. Grade 2 anemia was demonstrated in 2.5% in the 1^st^ week and 3.7% in the 3^rd^ week of treatment. Grade 2 leukopenia was found in 4.8% in the 2^nd^ week and increased to 9.8% in the 3^rd^ week. We also had grade 3 leukopenia in 1 patient (1.2%) in the last week of treatment. The incidence of severe grade hematologic toxicities was higher in the patient group who received RNI (4.2% *vs* 3.3% for grade ≥ 2 anemia and 16.7% *vs* 8.2% for grade ≥2 leukopenia). However, no statistically significant difference was demonstrated between these two groups. Due to the lack of reports on acute hematologic toxicities in most HF-SIB breast cancer studies, we were unable to compare our results. All studies (17–19) reported the acute skin toxicities but not the hematological toxicities. However, the incidence of severe grade hematologic toxicities in our study was very low and caused a delay of treatment in only 1 patient.

The integral dose to the whole body due to the large treatment volume of HF-WBI-SIB using HT has given concern for higher rates of acute hematologic toxicity. There is limited data about regarding the whole body integral dose for hypofractionated breast treatment by HT. Karpf et al. (20) compared the normal tissue integral dose (NTID) for tangential techniques between IMRT and VMAT. The IMRT technique significantly reduced NTID by 19% (p = 0.000005). Phurailatpam et al. (21) compared the whole-body integral dose for bilateral breast treatment between VMAT and HT. The whole-body integral dose was found to be comparable with no statistically significant variation between two techniques: 289 Gy kg (VMAT) versus 299 Gy kg (HT) (p-value 0.24). Our results reported a significantly higher whole body integral dose in the group of HF-WBI-SIB with RNI compared to the group without RNI (increase by 26.1%). Nevertheless, no statistically significant difference in hematologic toxicities was found between the two groups. Even though the higher whole body integral dose did not affect the acute toxicities, late toxicities should be close monitored in a long-term follow-up. We are also waiting for the report of acute and late toxicities and long-term outcomes in a large German phase III study comparing HF-WB-SIB to normal fractionation and/or sequential boosts (NCT02474641), enrolling more than 2,000 patients.

To the extent of our knowledge, even though there are some reports on HF-WBI-SIB, ours is the first study to explore HF-WBI-SIB with RNI. Moreover, we also investigated the whole-body integral dose and its association with acute hematologic toxicity. This phase II study did not compare HF-WBI-SIB to other SIB techniques, conventional fractionation, or other HF regimens, which can be considered one of its main limitations. Due to the short follow up time, we could not report the cosmetic outcome. The study is ongoing and we will address the treatment outcome, cosmesis, and late toxicities in a subsequent report.

## Conclusion

Based on our data, HF-WBI-SIB with or without RNI could be offered after breast conserving surgery and adjuvant chemotherapy. This scheme was feasible with acceptable acute skin and hematologic side effects.

## Data availability statement

The raw data supporting the conclusions of this article will be made available by the authors, without undue reservation.

## Ethics statement

The studies involving human participants were reviewed and approved by The Research Ethics Committee of Faculty of Medicine, Chiang Mai University, Ramathibodi Hospital, and Lopburi Cancer Hospital. The patients/participants provided their written informed consent to participate in this study.

## Author contributions

IC initiated and coordinated the study, interpreted the data, and wrote the manuscript. WN calculated the dose, analyzed the data, and wrote the manuscript. PS performed the statistical analysis. WO, PK, TN, CS, and SK performed clinical data acquisition. All authors contributed to the article and approved the submitted version.
